# Supplementary material for: Molecular and Functional Characterization of Pheromone Binding Protein 2 from Cyrtotrachelus buqueti (Coleoptera: Curculionidae)
Source: Int J Mol Sci. 2023 Nov 29;24(23):16925. doi: 10.3390/ijms242316925 (PMC10706763; doi:10.3390/ijms242316925)
Supplement: Supplementary file 1 [file ijms-24-16925-s001.zip › ijms-2702527-supplementary.pdf]

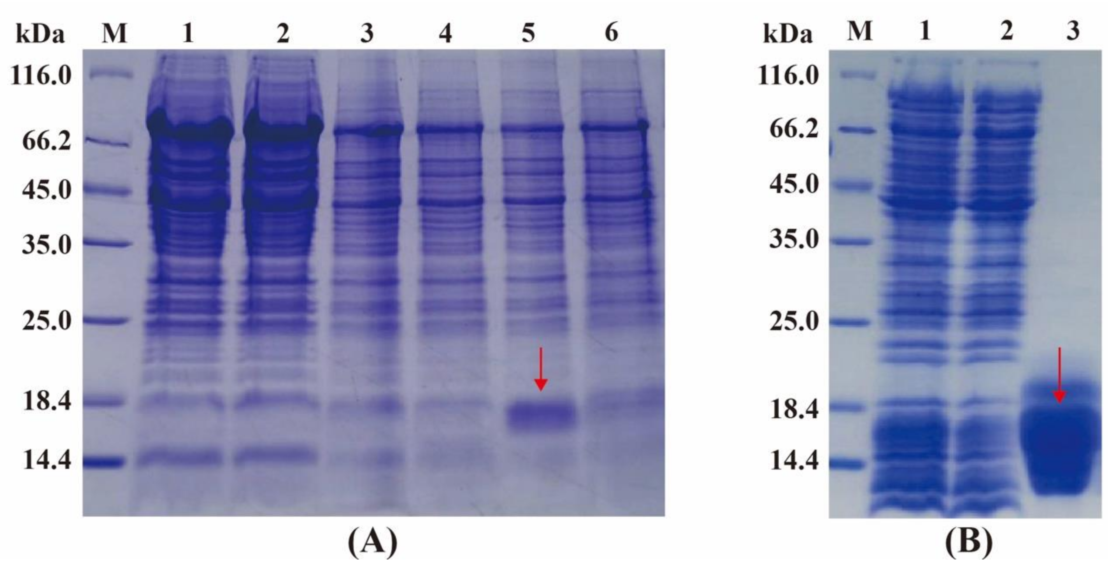

Figure S1. SDS-PAGE analysis of recombinant CbupPBP2 protein. **(A)** SDS-PAGE analysis of recombinant CbupPBP2 protein after induction with different IPTG concentrations. M: Protein marker; Lane 1: empty vector induced with 1 mM IPTG; 2-6: pET-28a (+)-CbuqPBP2 recombinant plasmid induced with 0 mM, 0.2 mM, 0.6 mM, 1.0 mM, 1.4 mM IPTG. **(B)** SDS-PAGE analysis of purified recombinant CbupPBP2 protein. M: protein marker; Lane 1: sonicated bacterial pellet; 2: protein in eluent; 3: purified protein.

Table S1. Primer sequences used in this study.

Primer name	Sequences (5′-3′) [a]
qPCR Primers for qPCR	
qPBP2-F	AACAATGCACGAACTGGAACG
qPBP2-R	AGCGTCTTGATATTCTTCTGGCA
qGAPDH-F	GGTCCATCCCACAAGGACTG
qGAPDH-R	AACCTTACCGACAGCCTTAGC
Primers for prokaryotic expression	
PBP2-F	TGGTGGACAGCAAATGGGTCGCGGATCCGAATTCGAGCTCGAT<br>GATGATGATAAAGATGAAATGAGAGAACTT
PBP2-R	AGCCGGATCTCAGTGGTGGTGGTGGTGGTGCTCGAGTT<br>AAATTAGGAAATAATGTTCCGGGCTTT
Primers for dsRNA synthesis	
dsPBP2-T7-F	**TAATACGACTCACTATAGG**ATGTTCAAGACTTTAACGATAGTTCTTACC
dsPBP2-R	TTAAATTAGGAAATAATGTTCCGGGCTTTC
dsPBP2-F	ATGTTCAAGACTTTAACGATAGTTCTTACC
dsPBP2-T7-R	**TAATACGACTCACTATAGG**TTAAATTAGGAAATAATGTTCCGGGCTTTC

[a] The sites for restriction digest in primers for prokaryotic expression are indicated by underscores. T7 promoter sequences in primers for dsRNA synthesis are in bold.
